# Clinical Efficacy Study of 755 nm Picosecond Laser Combined With 595 nm Pulsed Dye Laser in the Treatment of Port‐Wine Stain

**DOI:** 10.1111/phpp.70077

**Published:** 2026-03-10

**Authors:** Jian Huang, Yu Zhang, Zhen Tang, Binping Luo, Lina Tan, Jianyun Lu, Yaping Xiang, Lihua Gao

**Affiliations:** ^1^ Department of Dermatology, The Third Xiangya Hospital Central South University Changsha Hunan China

**Keywords:** efficacy assessment, picosecond laser, port‐wine stain, pulsed dye laser, sequential treatment

## Abstract

**Background:**

Port‐wine stain (PWS) is a disfiguring vascular anomaly characterized by persistent cutaneous erythema and progressive tissue hyperplasia. Although pulsed dye laser (PDL) is the first‐line treatment and alleviates certain clinical manifestations, incomplete lesion clearance and high recurrence rates persist in some patients, posing significant therapeutic challenges.

**Objective:**

To evaluate the efficacy and safety of sequential therapy with a 755 nm picosecond laser (PSL) combined with 595 nm PDL versus 595 nm PDL alone in treating PWS.

**Methods:**

Thirty‐four patients with PWS were enrolled. Lesions were randomly divided into paired subregions (PDL and PDL + PSL) using a subregional control design. Efficacy was assessed through objective and subjective measures: standardized clinical and dermoscopic images were used to quantify visual scores and clearance rates; reflectance confocal microscopy (RCM) quantitatively analyzed changes in vascular density and diameter; patient satisfaction and adverse reactions were also evaluated.

**Results:**

The PSL + PDL group showed significantly greater improvement in visual assessment scores, lesion area clearance, vascular density, vascular diameter, and patient satisfaction compared to the PDL group (all *p* < 0.05). While no statistically significant difference was observed in adverse event incidence rates between the two treatment modalities (*p* > 0.05), both regimens exhibited favorable safety profiles.

**Conclusion:**

The PSL + PDL regimen shows significantly superior efficacy over PDL alone, thus presenting a promising and advanced therapeutic alternative for PWS treatment.

Port‐wine stain (PWS) is a common congenital vascular malformation localized in the papillary dermis, with an estimated prevalence of 0.3%–0.5% among newborns. Irregular, non‐blanching, pinkish‐red patches of skin are present at birth. Over time, these lesions may progressively darken to a violet hue and become thickened or nodular in adulthood [1, 2]. Pulsed dye laser (PDL) is one of the most classic clinical treatments for PWS [3]. However, the efficacy of PDL monotherapy remains suboptimal in certain patients with PWS [4, 5], highlighting an imperative to develop more effective strategies.

Based on promising preliminary clinical observations, we propose that combination therapy with 755 nm picosecond laser (PSL) and PDL may significantly enhance treatment response. PSL is mainly utilized in pigmented, melasma, and scarring disorders and also promotes collagen regeneration and skin repair [6, 7], by virtue of its photomechanical effect, which enables more efficient destruction of target vessels and may theoretically produce complementary and synergistic effects with the photothermal mechanism of PDL. Although theoretically advantageous, this novel synergistic approach represents a promising yet underexplored therapeutic strategy that, to our knowledge, has not been systematically investigated in prospective controlled clinical studies comparing its efficacy and safety with conventional PDL monotherapy.

In this study, we aimed to evaluate the efficacy and safety of a novel sequential therapy combining PSL and PDL compared to conventional PDL monotherapy in patients with PWS. We conducted a comprehensive evaluation incorporating visual assessment, lesion area regression, vascular density, vessel diameter, patient satisfaction, and adverse event incidence rates. A total of 34 patients with PWS were enrolled from the Department of Dermatology at the Third Xiangya Hospital between January 2024 and December 2024. Each patient's lesions were randomly assigned to receive either sequential treatment with PSL + PDL or monotherapy with the 595 nm PDL alone, using a within‐patient comparative design.

## 1 Materials and Methods

### 1.1 General Information

This prospective, self‐controlled, paired trial included 34 patients with PWS, diagnosed by an experienced dermatologist, who were enrolled at the Third Xiangya Hospital between January 2024 and December 2024. The target sample size of 34 was set based on the anticipated number of eligible patients available for enrollment at our specialized center during the 12‐month study period. The cohort consisted of 24 females and 10 males, with ages ranging from 1 month to 56 years (mean ± standard deviation: 16.87 ± 15.40 years). Participants were stratified into the following age groups: 7 infants (0–3 years), 8 preschool and school‐age children (4–11 years), 7 adolescents (12–18 years), and 12 adults (> 18 years). Lesions were predominantly located on the face, neck, and limbs. The study protocol was approved by the Ethics Committee of our institution (Approval No.: Fast 24995), and written informed consent was obtained from all patients or their parents.

### 1.2 Treatment

Both the treatment center and operating doctors met predefined eligibility criteria to ensure treatment consistency. Lesions in each patient were bisected symmetrically into two areas, which were randomly designated as A and B. Among them, area A was treated with PSL + PDL, beginning with the PSL followed immediately by PDL. The random allocation sequence was generated using a computer‐based random number generator with a 1:1 allocation ratio. Treatments were administered at 4‐week intervals, with the total number of sessions (ranging from 3 to 9) tailored based on individual therapeutic response and lesion characteristics. Area B was treated with PDL, with the same treatment frequency and duration as that in area A. Posttreatment cold compresses were applied to mitigate discomfort and reduce swelling. Topical lidocaine ointment was used prior to each session for analgesia. Laser parameters and treatment counts were adjusted according to skin type and anatomic location of the lesion. No interim analyses were planned. Stopping guidelines mandated the immediate discontinuation of treatment for any patient experiencing a serious adverse event deemed related to the intervention, with appropriate supportive care provided. Standardized clinical photographs, dermoscopy, reflectance confocal microscopy (RCM), patient satisfaction, and adverse reaction data were collected promptly after treatment. Participants and outcome assessors were blinded to the allocation. The PSL treatment was delivered using a PicoSure system (Cynosure, USA), with a wavelength of 755 nm, a spot diameter of 6 mm, an energy density of 0.71 J/cm^2^. The 755‐nm PSL was delivered at a pulse density of 10 pulses/cm^2^. Each treatment area received two passes, resulting in a total cumulative pulse count of 20 pulses/cm^2^ per lesion. We used the zoom handpiece for all PSL treatments in this study. The PDL treatment was performed with a Vbeam system (Candela, USA), wavelength 595 nm, pulse with 0.45–1.5 ms, spot diameter 7 mm, energy density 8.0–10.0 J/cm^2^, synchronized DCD parameter set to 30 ms cooling time. No important changes were made to the trial methods, outcomes, or statistical analysis plan after the trial commenced.

### 1.3 Evaluation of Efficacy

#### 1.3.1 Visual Assessment

Treatment efficacy was assessed by comparing standardized clinical photographs taken before and after the intervention. The grading criteria were as follows: Excellent (lesion clearance ≥ 75%, score: 5); good (lesion clearance 51%–75%, score: 4–5); moderate (lesion clearance 25%–50%, score: 3–4); poor (lesion clearance < 25%, score: 2–3); basically no regression (lesion clearance ≤ 5%, score: 1–2). The relative lesion area was quantified from photographs using Image J software (National Institutes of Health, USA), and the clearance rate was calculated accordingly.

#### 1.3.2 Dermoscopy

Dermoscopic imaging was performed to evaluate the color of skin lesions, vascular morphology, and structural changes for dynamic monitoring of therapeutic efficacy. Images were acquired using a DermLite DL4 (Dermlite, USA) with 10× magnification.

#### 1.3.3 RCM

RCM compared the efficacy of the two treatments by obtaining vessel density and vessel diameter data. RCM device was with an imaged Vivascope 1500 system (Lucid Inc., USA) using a laser with a wavelength of 830 nm as a light source and a laser power of < 35 mW at the tissue level.

#### 1.3.4 Security Assessment

Adverse reactions were recorded after each treatment, with close monitoring for hyperpigmentation, blistering, crusting, edema, and other potential responses. The incidence rates of each adverse reaction were calculated, and differences in adverse event rates between the two treatment methods were statistically compared. Treatment safety was subsequently evaluated based on these findings.

#### 1.3.5 Patient Satisfaction

Patient satisfaction was evaluated using a five‐point scale consisting of the following grades: complete improvement, significant improvement, moderate improvement, slight improvement, and no improvement. The percentage of each grade was calculated separately to compare whether there was a difference in patient satisfaction between the two treatment groups.

### 1.4 Statistical Methods

SPSS 26 and GraphPad Prism10 statistical software were applied for statistical analysis. A paired *t*‐test was used to deal with visual assessment, degree of clearance of skin lesion area, vascular density, and vascular tube diameter in PSL + PDL and PDL, and the McNemar‐Bowker test was used to analyze the adverse reactions and patient satisfaction, with the test significance level of *α* = 0.05. Missing data were not imputed. The primary analysis was performed on a complete‐case basis, including only participants with outcome data available at all scheduled time points.

## 2 Results

All 34 enrolled patients received the allocated intervention and were included in the analysis. Treatments were administered at 4‐week intervals, with the total number of sessions (ranging from 3 to 9) tailored based on individual therapeutic response and lesion characteristics. No patients were lost to follow‐up or excluded from the study after randomization. Patients did not receive any concomitant therapies for PWS during the trial.

### 2.1 Visual Assessment

**FIGURE 1:**
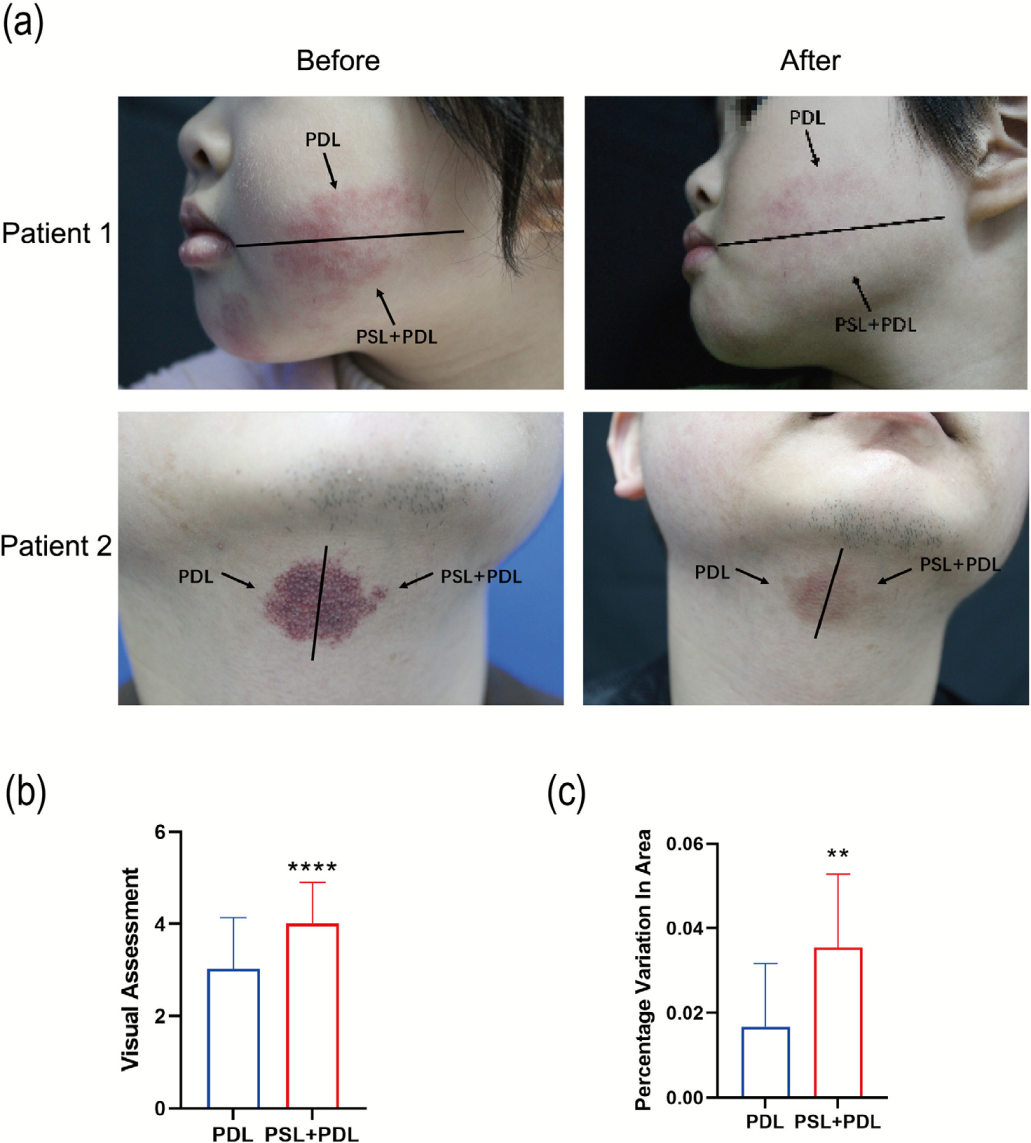
(a) Photographs of skin lesions before and after treatment by both methods in two patients. (b) Statistical graph of visual assessment (*n* = 15). (c) Statistical graph of percentage change in the area after treatment (*n* = 6). **p < 0.01, ****p < 0.0001.

Following treatment, lesions in both the PDL monotherapy and PSL + PDL combination groups demonstrated fading, progressing to a pinkish discoloration. The lesion areas showed a progressive reduction, characterized by peripheral‐to‐central contraction. Additionally, hypertrophic lesions exhibited flattening and softening in texture compared to baseline. The border of the lesions became blurred and gradually overgrown with normal skin. Visually, the PSL + PDL combination therapy demonstrated superior lesional lightening, with treated areas approximating normal skin tone, and exhibited more pronounced size reduction compared to PDL monotherapy (Figure 1a). Quantitative visual assessment revealed a statistically significant difference between groups: the PDL group achieved a mean improvement score of 3.03 ± 1.11, whereas the PSL + PDL group attained 4.00 ± 0.89 (*p* < 0.0001, Figure 1b). Furthermore, digital morphometric analysis using Image J software quantified the percentage of area reduction, showing significantly greater lesion clearance in PSL + PDL (3.54% ± 1.17%) versus PDL alone (1.68% ± 1.49%, *p* < 0.01, Figure 1c).

### 2.2 Dermoscopy

**FIGURE 2:**
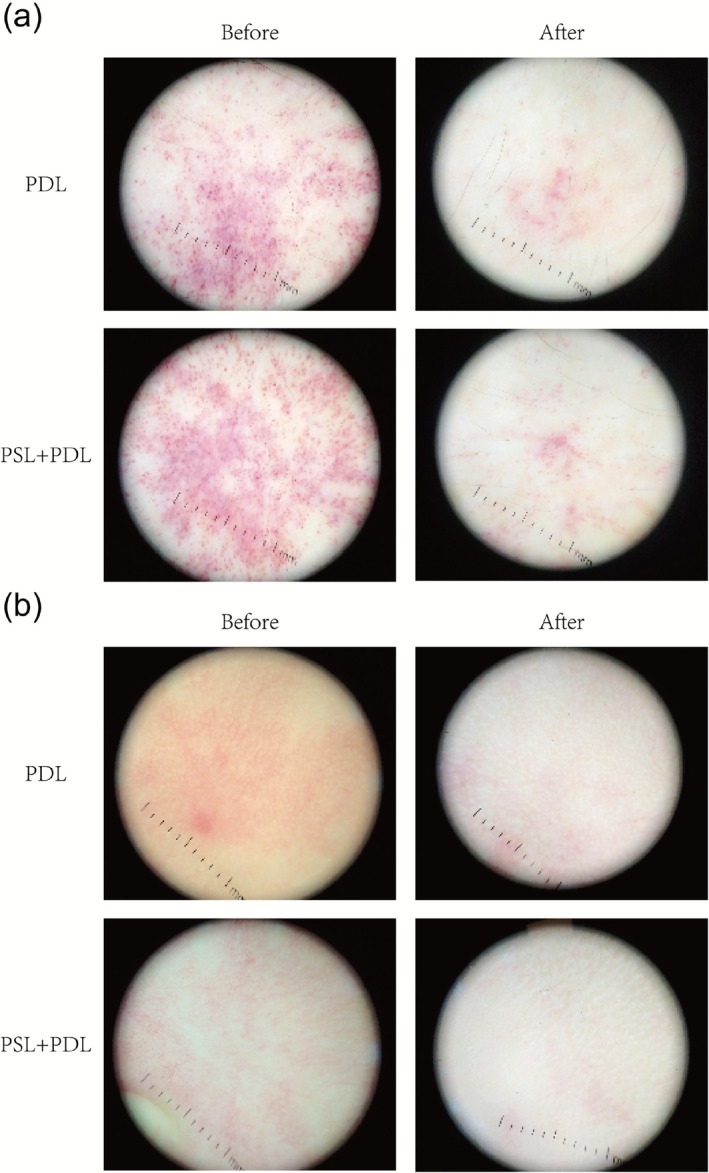
(a, b) Dermoscopic images of two patients before and after treatment with both methods of treatment.

Pretreatment dermoscopic images showed a dense distribution of short stippled and short linear vessels, with a high density of vessels on a purplish‐red background and no obvious areas of white fibrosis. After treatment, the background color became white, the original dense punctate blood vessels were significantly reduced, a small number of short linear blood vessels remained, and a little local discoloration was seen. A more pronounced reduction in vascular density was observed in the PSL + PDL‐treated areas compared to the PDL‐treated areas based on pre‐ and posttreatment evaluations (Figure 2a,b).

### 2.3 RCM

**FIGURE 3:**
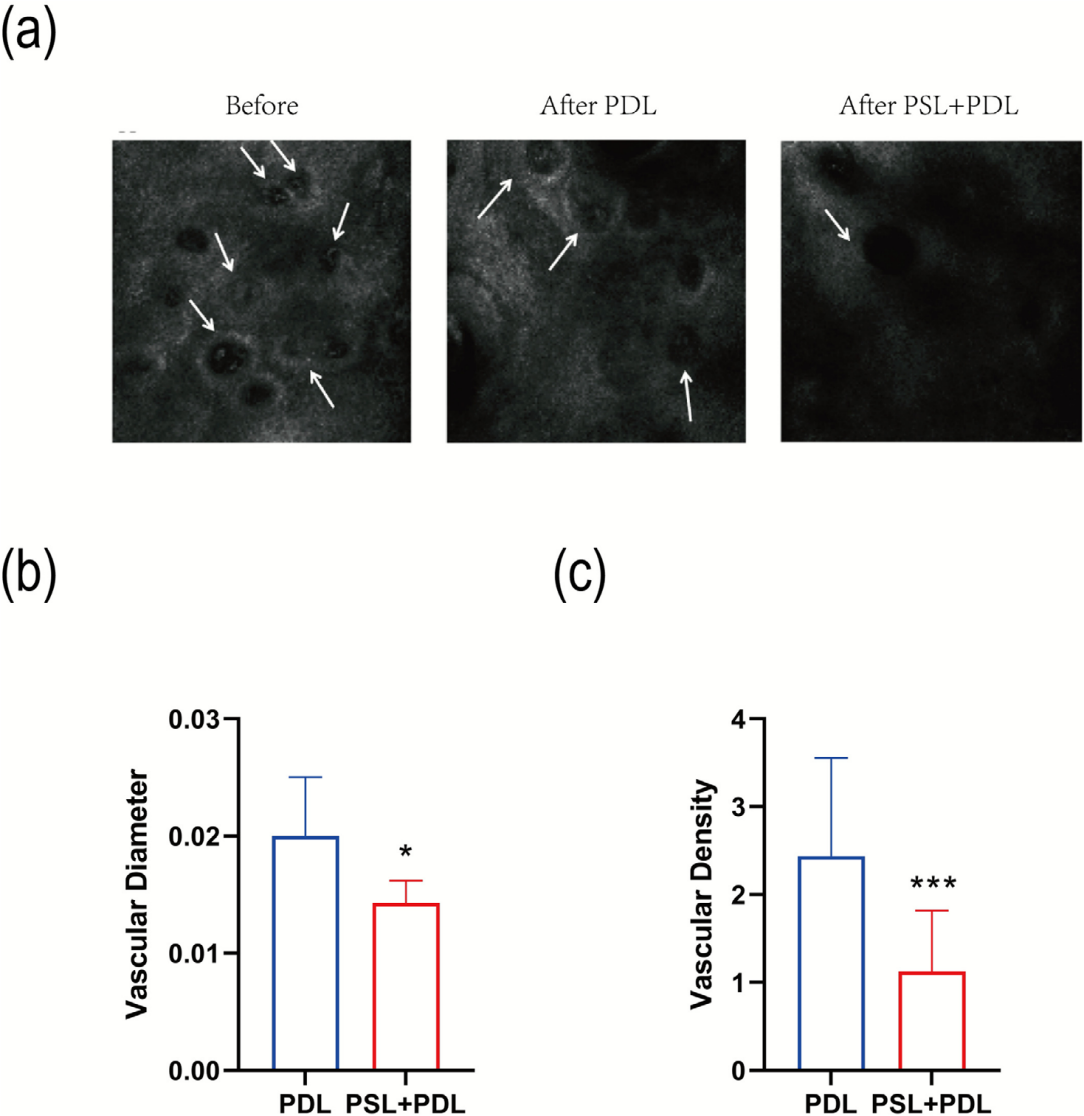
(a) RCM images of patients before treatment, after PDL treatment, and after PSL + PDL treatment. (b) Statistical plots of vessel diameter (*n* = 7) under RCM, respectively. (c) Statistical plots of vessel density (*n* = 8) under RCM, respectively. *p < 0.05, ***p < 0.001.

RCM examination revealed marked vascular hyperplasia with dilatation and congestion in the dermal papillae prior to treatment, surrounded by abundant perivascular inflammatory infiltrates and siderophages. After three sessions of PSL + PDL combination therapy, the basal layer pigmentation showed greater reduction compared to PDL treatment alone, with thinner skin thickness and deeper effective scanning depth. A more pronounced decrease in vascular density per unit area was observed in the PSL + PDL group versus PDL monotherapy (Figure 3a). Posttreatment measurements demonstrated significantly smaller vascular diameters in the PSL + PDL group (0.14 ± 0.02 mm) than in the PDL group (0.20 ± 0.05 mm) (*p* < 0.05, Figure 3b). The vascular density was markedly lower in the PSL + PDL group (1.13 ± 0.69 vessels/mm^2^) versus the PDL group (2.44 ± 1.12 vessels/mm^2^) (*p* < 0.01, Figure 3c). Quantitative analysis revealed a 46.41% reduction in vascular diameter and a 61.05% decrease in vascular density for the PDL group, whereas the PSL + PDL group achieved superior outcomes with a 59.03% diameter reduction and 82.20% density reduction.

### 2.4 Safety Assessment

Minor adverse events including postinflammatory hyperpigmentation, blisters, crusting, edema, and mild pain were occasionally observed in both treatment groups, all demonstrating self‐resolving characteristics with low incidence rates. Among 34 patients (192 treatment sessions analyzed in total), postinflammatory hyperpigmentation and blisters constituted the predominant adverse events, with incidence rates of 4.69% each in the PDL group, compared to 4.69% and 3.13% respectively in the PSL + PDL group. Edema and mild pain demonstrated lower occurrence frequencies. The total adverse event rates were 15.63% for PDL and 12.50% for PSL + PDL. McNemar‐Bowker test analysis of this paired multinomial categorical dataset revealed no statistically significant differences in adverse event distributions between groups (*p* > 0.05, Table 1, Table S1). Both therapeutic modalities exhibited favorable safety profiles characterized by low incidence rates, mild transient reactions, and spontaneous resolution without medical intervention.

**TABLE 1:** Adverse reaction statistics (*n* = 192).

	PDL	PSL + PDL	*p*
No adverse reaction	165 (85.94%)	168 (87.50%)	0.22
Hyperpigmentation	9 (4.69%)	9 (4.69%)	
Blisters	7 (3.65%)	5 (2.60%)	
Crust	5 (2.60%)	5 (2.60%)	
Edema	3 (1.56%)	2 (1.04%)	
Slight pain	3 (1.56%)	3 (1.56%)	
In total	192 (100%)	192 (100%)	

### 2.5 Patient Satisfaction

A standardized satisfaction survey encompassing five clinical response categories (complete improvement, significant improvement, moderate improvement, slight improvement, and no improvement) was conducted among 34 patients. McNemar‐Bowker testing of the proportional distributions revealed significant intergroup differences in satisfaction outcomes (*p* < 0.05). The PSL + PDL group demonstrated superior response profiles: 47.05% significant improvement, 29.41% moderate improvement, and 23.53% slight improvement, compared to 29.41%, 32.35%, and 38.24% respectively in the PDL group. Notably, neither cohort reported complete resolution or treatment failure. Comparative analysis of improvement gradients indicates overall higher patient satisfaction with PSL + PDL therapy versus PDL monotherapy (Table 2, Table S2).

**TABLE 2:** Patient satisfaction (*n* = 34).

Groups	PDL	PSL + PDL	*p*
Complete resolution	0 (0)	0 (0)	0.03*
Significant improvement	10 (29.41%)	16 (47.05%)	
Moderate improvement	11 (32.35%)	10 (29.41%)	
Slight improvement	13 (38.24%)	8 (23.53%)	
No improvement	0 (0)	0 (0)	

* *p* < 0.05.

## 3 Discussion

PWS are flat, pinkish lesions in infancy that do not fade, and as the disease progresses further, the lesions thicken and deepen in color to purple [8]. PWS located in visible areas adversely affects the mental health of patients and their families [9]. Early treatment is critical to minimize the difficulty of treatment, improve outcomes, and reduce psychological stress [10, 11]. There are a variety of treatments available for PWS, and the choice of treatment should be based on the type, location, and severity of the lesion, as well as individual patient characteristics. Among these, PDL remains the primary clinical option, demonstrating optimal efficacy with minimal adverse effects and a well‐established safety profile [12]. The therapeutic efficacy of PDL in PWS is mediated through selective photothermolysis [13], wherein laser energy selectively targets hemoglobin, inducing thermal coagulation of the vascular walls and surrounding dermis [14, 15]. The extended pulse duration combined with integrated epidermal cooling mechanisms in PDL systems preserves epidermal integrity while minimizing collateral thermal damage. The long pulse duration and epidermal cooling techniques of PDL effectively protect the epidermis, minimize thermal damage, and ensure sufficient heat delivery to target blood vessels, thereby enhancing therapeutic efficacy.

This study demonstrates that the sequential combination therapy of 755 nm PSL and 595 nm PDL is significantly superior to conventional PDL monotherapy in treating PWS, with regard to lesion clearance rate, improvement in vascular parameters, and patient satisfaction, while exhibiting a comparable safety profile. Visual assessments, lesion area regression, and patient‐reported outcome measures consistently demonstrated superior efficacy in the PSL + PDL cohort compared to PDL monotherapy, with statistically significant intergroup differences (*p* < 0.05). Objective data from RCM further confirmed the enhanced therapeutic effect of combination therapy, demonstrating greater reductions in both vascular density and vessel diameter in the PSL + PDL group relative to the PDL‐alone group. In addition, both groups showed a high safety profile with a low incidence of adverse effects.

Indeed, PDL remains the golden standard for PWS treatment by selectively targeting oxyhemoglobin within ectatic vessels. Concurrently, PSL has gained recognition in managing postinflammatory conditions characterized by capillary dilation, such as postinflammatory erythema and postinflammatory hyperpigmentation [16]. It is evident that the PDL and PLS combination treatment is effective in aiding a greater number of patients with PWS. The efficacy of PSL is largely attributable to its potent photoacoustic effect, which induces direct vascular destruction and physical remodeling of the perivascular dermal matrix and collagen network. This remodeling leads to increased tissue density and enhanced mechanical support around the vessels [17]. As the shockwave propagates through the dermal, it delivers controlled micro‐injuries to collagen fibrils. These micro‐injuries serve as a potent initiating signal for the wound healing cascade, upregulating cytokines and growth factors that prepare the tissue for subsequent collagen synthesis and remodeling [18]. In the context of PWS, this process results in a restructured and tightened perivascular stroma. The dilated vessels become embedded in a denser, more mechanically supportive microenvironment. Such PSL‐induced stromal densification creates critical spatial and mechanical constraints that enhance the final PDL treatment. In summary, the proposed synergy is not merely additive but sequential and logical: the PSL acts as a “physical preconditioning” agent that directly perturbs vessels, switches the dermal microenvironment to a proactive remodeling state, and finally constructs a confining scaffold that maximizes and sustains the vascular destructive effect of the gold‐standard PDL.

The superior efficacy observed in the combination therapy group may also be attributable to the complementary mechanisms of action between PSL and PDL. Through mathematical models and animal experiments, researchers have found that the optimal treatment wavelength for PDL becomes longer as the melanin content of the epidermis increases, suggesting that melanin in the skin preferentially absorbs the energy of the laser [19]. During PDL treatment, the main target of the laser is hemoglobin, but the presence of melanin interferes with the selective action of the laser on the diseased blood vessels, resulting in the dispersion of the energy, which cannot effectively act on the target blood vessels, thus decreasing the therapeutic effect. PSL is mainly applied to pigmented diseases [20], which can effectively reduce the melanin in the skin as well as postinflammatory discoloration produced by simple PDL treatment, thus reducing the limitations of PDL treatment. However, the melanin competition mechanism has not yet been substantiated by direct experimental evidence. Further studies are required to elucidate the precise underlying mechanism.

Despite PDL being the gold standard for PWS, clinical outcomes remain suboptimal in a substantial proportion of patients, particularly those with hypertrophic lesions where complete clearance is seldom achieved, underscoring the inherent limitations of PDL monotherapy [21]. This therapeutic gap necessitates the exploration of multimodal strategies. Several combination approaches have been investigated. For instance, the addition of anti‐angiogenic agents such as rapamycin—which modulates vascular endothelial growth factor expression—has been shown to reduce reperfusion rates and improve efficacy when combined with PDL [22]. Other researchers combined PDL and a beta‐blocker (timolol gel) to treat PWS but did not significantly reduce disease recurrence [23]. In another study, 10 patients with recalcitrant PWS were treated with a PDL combined with radiofrequency (RF), and good outcomes were obtained [24]. Alster et al. further reported improved efficacy in recalcitrant and hypertrophic PWS using dual‐wavelength sequential irradiation (595 nm PDL + 1064 nm Nd: YAG laser) [25]. There are various combination treatment options for PWS, including laser combined with phototherapy and drug combined with laser therapy. However, in contrast to the studies mentioned above, the present work not only provides the first systematic theoretical rationale for combining PSL with PDL therapy but also offers stronger clinical evidence through a prospective controlled trial, convincingly demonstrating the superiority and clinical translational potential of this combined strategy for refractory PWS.

This study is the first not only to establish a novel theoretical rationale for the combination of PSL and PDL therapy, but also to provide robust prospective controlled data demonstrating its superiority over gold‐standard PDL monotherapy. A key innovation of this work lies in the development and validation of a new treatment strategy grounded in mechanistic synergy. In contrast to the purely photothermal effects of conventional PDL therapy, we systematically implemented—for the first time—a sequential combination of the photomechanical effect of a PSL and the photothermal effect of a PDL. This work demonstrates the synergistic interplay between photomechanical fragmentation and photothermal coagulation, thereby establishing a novel theoretical framework for achieving more complete vascular clearance. Moreover, we implemented a multimodal imaging assessment system—including quantitative analysis of vessel density and diameter via RCM—thereby providing objective and precise microscopic evidence for therapeutic evaluation, an approach that has seldom been employed in previous studies.

This study also has several limitations. Although the sequential PSL + PDL therapy demonstrated significant superiority over PDL monotherapy (*p* < 0.05), the limited sample size (*n* = 34) restricts the precision of the treatment effect estimates—such as confidence intervals—and the ability to assess the risk of infrequent adverse events. Future multicenter randomized controlled trials with larger cohorts that include patients across diverse skin types and lesion characteristics are needed to validate these findings and improve their generalizability. Furthermore, the absence of long‐term follow‐up limits our ability to evaluate the durability of the treatment outcomes. Furthermore, this study did not include a PSL‐only treatment arm or a reversed sequence (PDL → PSL) group. Therefore, the independent effect of PSL and the potential influence of treatment sequence could not be determined. These aspects should be explored in future controlled investigations.

In conclusion, the sequential PSL + PDL therapy represents an effective and safe strategy that is superior to conventional PDL monotherapy for the treatment of PWS. These findings support its incorporation into clinical practice as a promising treatment option, particularly for cases resistant to standard laser therapy. Looking forward, future investigations should focus on conducting larger multicenter randomized controlled trials with extended follow‐up periods to further validate these results. Moreover, subsequent studies ought to explore optimized personalized treatment parameters and utilize noninvasive imaging technologies, such as RCM, to elucidate the underlying mechanistic details of the combination therapy.

## Funding

This work was supported by the Science and Technology Department of Tibet Autonomous Region (no. XZ202402ZY0002), Natural Science Foundation of Hunan Province (2025JJ80143).

## Ethics Statement

The study protocol was approved by the Ethics Committee of the Third Xiangya Hospital (approval number: Fast 24995).

## Consent

All patients or their parents had signed a written informed consent.

## Conflicts of Interest

The authors declare no conflicts of interest.

## Supporting information


**Tables S1–S2:** phpp70077‐sup‐0001‐TablesS1‐S2.docx.

## Data Availability

The data that support the findings of this study are available from the corresponding author upon reasonable request.
