# Supplementary material for: Clinical Efficacy Study of 755 nm Picosecond Laser Combined With 595 nm Pulsed Dye Laser in the Treatment of Port‐Wine Stain
Source: Photodermatol Photoimmunol Photomed. 2026 Mar 10;42(2):e70077. doi: 10.1111/phpp.70077 (PMC12976183; doi:10.1111/phpp.70077)
Supplement: Supplementary file 1 — Tables S1–S2: phpp70077‐sup‐0001‐TablesS1‐S2.docx. [file PHPP-42-e70077-s001.docx]

**Supplementary Tables**

**Table S1** Pairwise Comparison of Treatment Response (n=192)

|  | | PSL+PDL | | | | | |  |
| --- | --- | --- | --- | --- | --- | --- | --- | --- |
| PDL |  | no adverse reaction | hyperpigmentation | blisters | crust | edema | slight pain | in total |
|  | no adverse reaction | 165 | 0 | 0 | 0 | 0 | 0 | 165 |
|  | hyperpigmentation | 0 | 9 | 0 | 0 | 0 | 0 | 9 |
|  | blisters | 2 | 0 | 5 | 0 | 0 | 0 | 7 |
|  | crust | 0 | 0 | 0 | 5 | 0 | 0 | 5 |
|  | edema | 1 | 0 | 0 | 0 | 2 | 0 | 3 |
|  | slight pain | 0 | 0 | 0 | 0 | 0 | 3 | 3 |
| in total | | 168 | 9 | 5 | 5 | 2 | 3 | 192 |

**Table S2** Paired Comparison of Patient Satisfaction (n=34)

|  | | PSL+PDL | | | | | |
| --- | --- | --- | --- | --- | --- | --- | --- |
| PDL |  | complete resolution | significant improvement | moderate improvement | slight improvement | no improvement | in total |
|  | complete resolution | 0 | 0 | 0 | 0 | 0 | 0 |
|  | significant improvement | 0 | 6 | 7 | 0 | 0 | 13 |
|  | moderate improvement | 0 | 2 | 2 | 7 | 0 | 11 |
|  | slight improvement | 0 | 0 | 1 | 9 | 0 | 10 |
|  | no improvement | 0 | 0 | 0 | 0 | 0 | 0 |
| in total | | 0 | 8 | 10 | 16 | 0 | 34 |
